# Comparison of the Retinal Straylight in Pseudophakic Eyes with PMMA, Hydrophobic Acrylic, and Hydrophilic Acrylic Spherical Intraocular Lens

**DOI:** 10.1155/2014/340759

**Published:** 2014-03-03

**Authors:** Ya-wen Guo, Jun Li, Hui Song, Xin Tang

**Affiliations:** Tianjin Eye Hospital, Tianjin Key Laboratory of Ophthalmology and Visual Science, Clinical College of Ophthalmology, Tianjin Medical University, Tianjin Institute of Ophthalmology, Tianjin 300020, China

## Abstract

*Purpose*. To investigate the intraocular straylight value after cataract surgery. *Methods*. In this study, 76 eyes from 62 patients were subdivided into three groups. A hydrophobic acrylic, a hydrophilic acrylic, and a PMMA IOL were respectively, implanted in 24 eyes, 28 eyes, and 24 eyes. Straylight was measured using C-Quant at 1 week and 1 month postoperatively in natural and dilated pupils. *Results*. The hydrophilic acrylic IOLs showed significantly lower straylight values than those of the hydrophobic acrylic IOLs in dilated pupils at 1 week and 1 month after surgery (*P* < 0.05). However, the straylight values of the hydrophilic acrylic IOLs were the lowest among the three IOL groups. No significant difference was observed in straylight between 1 week and 1 month postoperatively in each group with natural and dilated pupils (*P* > 0.05). Moreover, no significant difference was found in straylight between natural and dilated pupils in each group at 1 week and 1 month postoperatively (*P* > 0.05). *Conclusions*. Although the hydrophobic acrylic IOL induced more intraocular straylight, straylight differences among the 3 IOLs were minimal. Pupil size showed no effect on intraocular straylight; the intraocular straylight was stable 1 week after surgery.

## 1. Introduction

Point spread function (PSF) is used to define the quality of a retinal image. When an eye is looking at a point source, the luminance distribution on the retina is called the PSF [1]. The visual acuity corresponds to the central part of the PSF (0~1 min of arc), and the outer part of the PSF (>1°) is the straylight. Straylight is caused by light that enters the eye and is scattered rather than focused on the retina. Straylight can create a veil of light over the retina, which can lead to halo, glare, hazy vision, and night blindness while driving [2, 3].

The cornea, iris, sclera, retina, and lens are the five major sources that contribute to intraocular straylight [4]. Changes in intraocular straylight after cataract surgery are mainly caused by intraocular lenses (IOLs). Although advances in IOL design have improved postoperative visual outcomes, patients often complain of reduced contrast and glare after cataract surgery. A recent study on European drivers showed large variations in straylight values in pseudophakic eyes [5]. Thus, visual acuity is not enough for evaluating visual function after cataract surgery. Recently, more attention has been directed at intraocular straylight. Estimating intraocular straylight is important in the evaluation of vision quality after cataract surgery [6].

The IOL material should be considered in studying the influence of intraocular straylight on pseudophakic eyes [7]. Different IOL materials may cause different amounts of intraocular straylight. This study aims to determine whether there are differences among PMMA, hydrophilic acrylic, and hydrophobic acrylic IOLs in perceived intraocular straylight. The light scattered from the edge of IOL is likewise important. Patients who underwent cataract extraction with IOL implantation may complain about dysphotopsia, entopic phenomena, and photic phenomena [8]. The optic edge has an important function in the occurrence of dysphotopsia [9]. Furthermore, we want to assess the differences among the various IOL materials when the pupil is dilated with the IOL edge exposed.

Straylight is difficult to measure in living eyes. The C-Quant straylight meter proposed by Van Den Berg offers a convenient technique that uses the compensation comparison method [10]. Moreover, Cerviño and Guber both showed that C-Quant possesses excellent reliability and high reproducibility [11, 12].

## 2. Materials and Methods

All the procedures followed the tenets of the Declaration of Helsinki and were approved by the local ethics committee. We certify that all applicable institutional and governmental regulations concerning the ethical use of human volunteers were followed during this research.

### 2.1. Participants

Seventy-six eyes of Sixty-two patients (age 64.86 ± 8.39 years, range 41–79; 38 women and 24 men) were included in this study. The patients were scheduled for phacoemulsification and IOL implantation from May 1, 2012, to December 31, 2012, in Tianjin Eye Hospital. Exclusion criteria were history of ocular surgery and ocular disease other than cataract.

### 2.2. Surgery

All the surgeries were performed by the same doctor (Ya-wen Guo). Phacoemulsification with a 3.2 mm clear corneal incision in the hydrophobic acrylic and hydrophilic acrylic group and a 5.5 mm Sclera tunnel incision in the PMMA group were followed by IOL implantation in the capsular bag. A hydrophobic acrylic spherical IOL (AR40e, AMO, USA) was implanted in the hydrophobic acrylic IOL group. A hydrophilic acrylic spherical IOL (HQ201hep, Hexavision, France) was implanted in the hydrophilic acrylic IOL group. A PMMA spherical IOL (PC156C55, Henan universe IOL R&M Co., China) was implanted in the PMMA group. The diameters of pupil were measured with wave-front analyzer KR-1W in dark room (Topcon, Tokyo Medical System Inc., Japan) with natural pupil and dilated pupil.

### 2.3. Straylight Measurement

The retinal straylight was measured with C-Quant straylight meter (Oculus Optikgeräte, GmbH, Wetzlar, Germany). It uses the compensation comparison method described by Franssen et al. [10]. It is more suitable for clinical examination than the instrument which works with direct compensation method. The center of the test field is divided in half. When the compensation light is presented to one-half, no compensation light is presented to the other. Outside the center is a ring-shaped flickering light source, which serves as the straylight source. When the subject is tested, one-half of the center has counter-phase flickering added, the other has not. The subject is asked to indicate the half which flickers stronger. The straylight meter will change the luminance of the stimulus and counter-phase modulating light automatically until the two halves are balanced. According to the subjects' responses, a psychometric function is defined from which the intraocular straylight value is obtained. All the subjects were examined with natural and dilated pupils one week and one month after surgery. The subjects were examined in dark and quiet room. Only when the estimated standard deviation (ESD) was lower than 0.08 and the quality factor (Q) was higher than 1.00, the measurement was accepted.

### 2.4. Statistical Analysis

All analyses were performed with SPSS 16.0 statistics software package (SPSS Inc., Chicago, Illinois). The straylight data were presented as the mean ± SD. The straylight values of three groups were compared with one-way analysis of variance (ANOVA). The LSD (least significance difference) was used to identify effects among the IOL types. In each pseudophakic group a paired *t*-test was used to compare the straylight values before and after pupil dilation. Comparison of the straylight one week and one month after surgery was performed with paired *t*-test. *P* < 0.05 was regarded as statistically significant.

## 3. Results

All pupils involved in this study were round with no iris trauma and showed good responsiveness to light after surgery. The IOLs were well centered and not tilted.

The mean ages of the hydrophobic acrylic, hydrophilic acrylic, and PMMA IOL groups were 67.17 ± 6.82, 62.82 ± 9.50, and 64.92 ± 8.16. There were no significant differences between the three groups (*P* > 0.05).

Table 1 shows the characteristics of the rigid PMMA, hydrophilic acrylic, and hydrophobic acrylic IOLs evaluated.

Table 2 shows the mean diameter of pupil one week after surgery and one month after cataract surgery. There were no statistically significant differences among groups 1 week after surgery and 1 month after surgery (*P* > 0.05).

Table 3 shows the descriptive statistics in three groups. The hydrophilic acrylic IOL showed a significantly lower straylight value than the hydrophobic IOL with dilated pupil one week after surgery (*P* = 0.002). A similar difference was found one month after surgery (*P* = 0.017). No significant difference was noted between other groups (*P* > 0.05). There was not a significant difference between hydrophilic acrylic and PMMA IOL group (*P* > 0.05). But the straylight values of hydrophilic acrylic IOL were the lowest of the three IOL groups.

**Figure 1:**
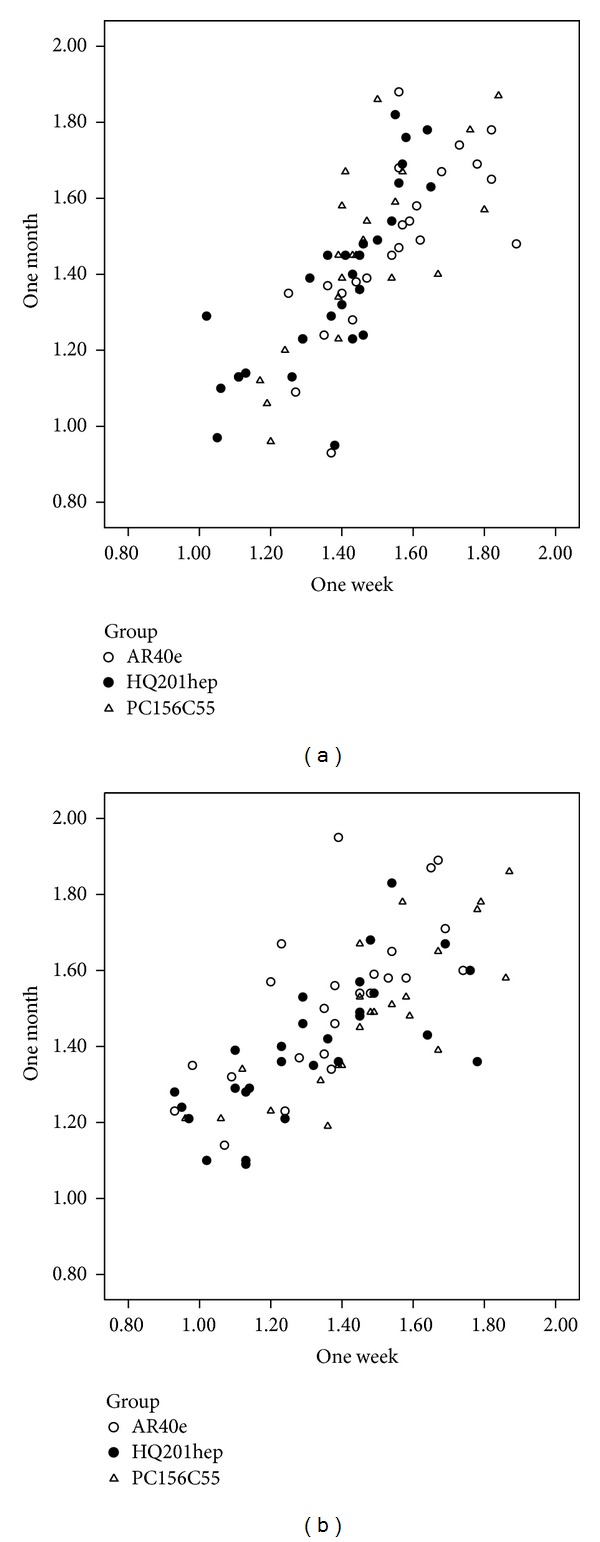
(a) The scatterplot of the straylight values of three IOLs 1 week after surgery (*x*-axis) and 1 month after surgery (*y*-axis) with natural pupil. (b) The scatterplot of the straylight values of three IOLs 1 week after surgery (*x*-axis) and 1 month after surgery (*y*-axis) with dilated pupil.

There was no significant difference in retinal straylight between one week and one month after surgery in each IOL group with natural pupil (*P* > 0.05). A similar result was found with dilated pupil (*P* > 0.05) (Figure 1).

**Figure 2:**
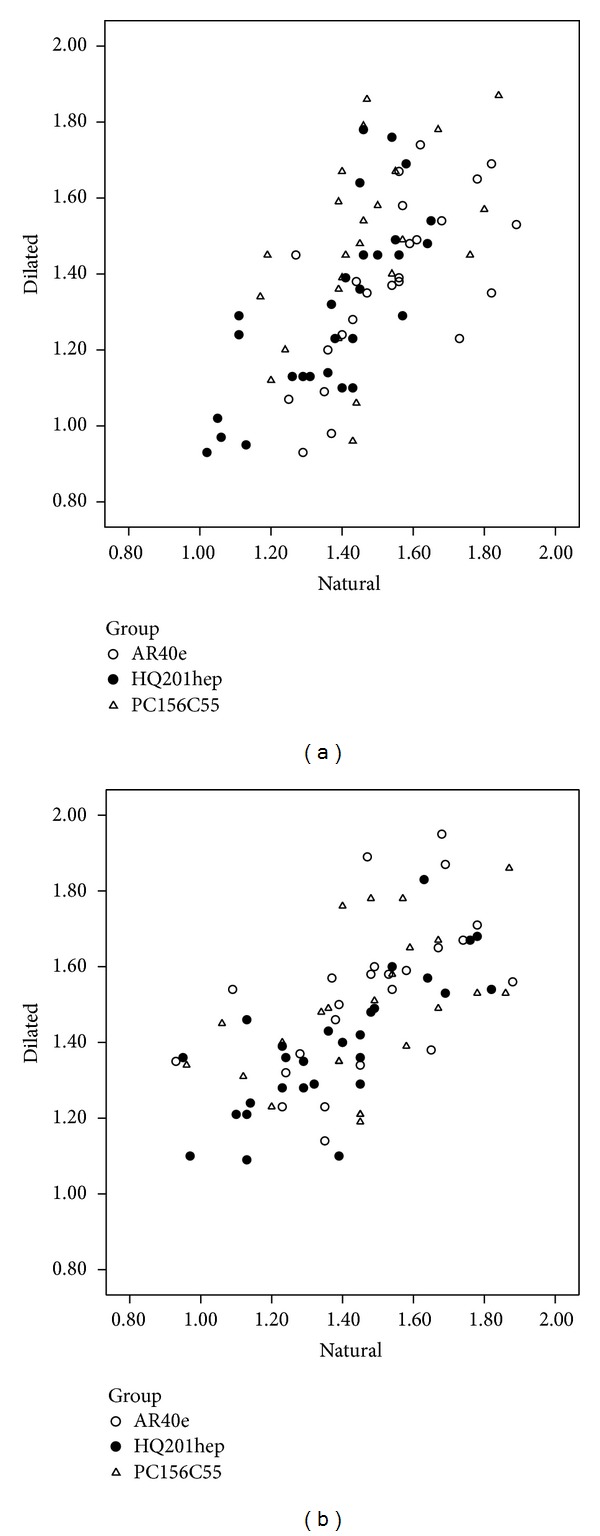
(a) The scatterplot of the straylight values of three IOLs before (*x*-axis) and after (*y*-axis) pupil dilation one week after surgery. (b) The scatterplot of the straylight values of three IOLs before (*x*-axis) and after (*y*-axis) pupil dilation one month after surgery.

No significant difference was found in retinal straylight between natural and dilated pupil in each IOL group 1 week after surgery (*P* > 0.05). A similar result was found a month after surgery (*P* > 0.05) (Figure 2).

## 4. Discussion

Patient complaints regarding dysphotopsia and glare after IOL implantations have recently gained a great deal of attention. Intraocular straylight is the major reason that results in such complaints. IOL is the main source of the increase in intraocular straylight after cataract surgery. Different IOL materials can cause various degrees of scatter. Thus, the IOL material should be taken into consideration. In this study, PMMA, hydrophilic acrylic, and hydrophobic acrylic IOLs were investigated. Only the hydrophilic acrylic IOLs showed a significantly lower straylight value than the hydrophobic acrylic IOLs in dilated pupils. No significant difference was found in straylight between the other groups. With or without dilated pupil, the straylight value of the hydrophilic acrylic IOL was the lowest among the three IOL materials. With natural pupil, the surface light scatter of IOL is the main source that induces the straylight. The hydrophilic acrylic IOL may induce less surface light scatter. Erie's study indicated that the sharp IOL edge could increase the light scatter. The square edge of IOLs can cause clinically significant glare and halo [13, 14]. The edge of the IOL may have been exposed when the pupil was dilated, thereby increasing light scatter. Accordingly, hydrophobic IOLs induced more scattering. Did the hydrophobic acrylic material induce more straylight? Further study in vitro should be needed. And the difference in the optic diameter between the PMMA IOL and the other two IOLs should theoretically change the straylight value with dilated pupils. More study is recommended.

Montenegro et al. [7] also found that postoperative straylight in the hydrophilic acrylic group was lower than that of the hydrophobic acrylic group. He supposed that the glistenings around the hydrophobic acrylic IOL can explain the reason for the higher amount of straylight induced by the hydrophobic acrylic IOLs. When IOLs are implanted into the eyes, some fluid-filled microvacuoles form in the hydrophobic IOL optics, which are called glistenings [15]. The glistenings were observed from as early as one week after surgery until after several months [16, 17]. The high incidence of glistenings was associated with hydrophobic acrylic IOLs, with an incidence of 55% at one year after the hydrophobic acrylic IOL implantation. A Japanese study showed that internal light scattering caused by the glistenings within the optic of the AcrySof IOLs increased over time up to three years after surgery [15]. The high incidence of glistenings may be the reason for the higher straylight values in the hydrophobic acrylic IOL group. In the present study, we found that the straylight values at one week and one month after hydrophilic acrylic IOL surgery were both lower than those of the hydrophobic acrylic IOLs. The formation of glistenings requires a longer time. In addition, we did not find any apparent glistening in the hydrophobic acrylic IOL using a slit lamp.

In the present study, we did not find an increase in straylight values with increasing pupil size at one week and one month after surgery. Franssen's study showed that there was no significant difference in straylight between pupils with diameters of 2.0 mm and 7.0 mm of healthy phakic eyes. They supposed that the large pupils increased the proportion of scattered light. The same results were found in the proportion of nonscattered light. The straylight values were unchanged [18]. Cerviño et al. also found that straylight values were not associated with the pupil size of the subject [19]. However, van Gaalen et al. reported that straylight increased with increasing pupil size in pseudophakic eyes at one year after surgery [20]. Straylight values were significantly higher in dilated than those in natural pupils, which was contradictory to our study. In van Gaalen's study, the presence of the anterior capsule rim in the pupillary area, not the pupil size, was considered as the main reason for the increase in straylight. The opacity of the anterior capsule rim may have increased the straylight significantly [20]. van Gaalen measured straylight using natural pupils and dilated pupils at one year after surgery. Although the eyes that had posterior capsular opacification (PCO) within the 3.0 mm diameter central zone of the posterior capsule were excluded, the migration of residual lens epithelial cells may have resulted in the opacification of the peripheral anterior and posterior capsules. This mechanism may increase straylight. In this study, we did not find PCO in any of the patients during the brief follow-up period. The follow-up period of one month is not enough to elicit changes of light scatter related to anterior capsule opacification. Thus, a long observation period is recommended.

In the early postoperative period, subclinical corneal edema supposedly increased straylight values [20]. Elliott et al. found an increase in straylight with increasing corneal thickness [21]. They thought corneal edema could increase straylight values. In the present study, we did not find any marked cornea edema in all the subjects using slit lamp. But corneal thickness was not measured. The corneal pachymetry should be used in further study. No significant differences were found in straylight values in natural pupils at one week and one month after surgery; similar results were obtained in dilated pupils. Thus, the straylight values in pseudophakic eyes have stabilized one week after surgery. van der Meulen et al. found no significant differences in straylight values between the patients examined within 30 days after surgery and those examined after 30 days or longer postoperatively. He reported that postoperative corneal edema does not affect intraocular straylight [22]. We think corneal edema increase straylight. In this study and van der Meulen's study all subjects were measured 7 and 30 days after surgery. It was long enough for corneal edema to disappear.

In the present study, we measured the intraocular straylight using the highly reproducible C-Quant [12]. Two parameters (ESD and Q) were used to determine the reliability of the measurement. The measurement results are considered reliable when the ESD is lower than 0.08 and the Q is higher than 1. Our measurement results satisfied the standard although all our patients were elderly individuals. Moreover, old age did not affect the measurement reliability of C-Quant using the compensation comparison method [11].

## Figures and Tables

**Table 1:** Characteristics of the rigid PMMA, hydrophilic acrylic, and hydrophobic acrylic IOLs evaluated.

	PC156C55	AR40e	HQ201hep
Diameter of optics (mm)	5.5	6.0	6.0
Shape	Biconvex	Biconvex	Biconvex
Material of optics	UV-absorbing PMMA	UV-absorbing hydrophobic acrylic	UV-absorbing hydrophilic acrylic
Surface	Spherical	Spherical	Spherical
Estimated *A*-constant	118.2	118.4	118.2
Refractive index	1.49	1.47	1.46
Abbe number	57	55	60
Haptic material	PMMA	PMMA	Hydrophilic acrylic
IOL type	1 piece	3 pieces	1 piece
Length (mm)	12.5	13	12.5

PMMA: polymethylmethacrylate, IOL: intraocular lens, UV: ultraviolet light.

**Table 2:** The mean diameter of pupil 1 week after surgery and 1 month after surgery.

IOL type	The diameter of pupil (mean ± SD, mm)			
	1 week after surgery		1 month after surgery	
	Natural pupil	Dilated pupil	Natural pupil	Dilated pupil
AR40e	3.85 ± 0.27	7.11 ± 0.38	3.87 ± 0.29	7.16 ± 0.33
HQ201hep	3.84 ± 0.26	7.03 ± 0.31	3.84 ± 0.20	7.00 ± 0.37
PC156C55	3.87 ± 0.25	7.12 ± 0.35	3.78 ± 0.27	7.14 ± 0.46
*F*	0.08	0.49	0.69	1.57
*P*	0.92	0.62	0.50	0.22

SD: standard deviation.

**Table 3:** Straylight values of pseudophakic eyes with hydrophobic acrylic, hydrophilic acrylic, and rigid PMMA IOLs.

IOL type	Straylight log(s)			
	1 week after surgery		1 month after surgery	
	Natural pupil	Dilated pupil	Natural pupil	Dilated pupil
Hydrophobic acrylic (AR40e) (*n* = 24)	1.46 ± 0.25 (0.84 to 1.86)	1.54 ± 0.18 (1.25 to 1.89)	1.47 ± 0.22 (0.93 ± 1.88)	1.53 ± 0.21 (1.14 to 1.95)
Hydrophilic acrylic (HQ201hep) (*n* = 28)	1.42 ± 0.20 (0.99 to 1.89)	1.38 ± 0.18 (1.02 ± 1.65)	1.37 ± 0.24 (0.95 to 1.82)	1.39 ± 0.18 (1.09 to 1.83)
PMMA (PC156C55) (*n* = 24)	1.48 ± 0.14 (1.24 to 1.70)	1.46 ± 0.18 (1.17 to 1.84)	1.45 ± 0.23 (0.96 to 1.87)	1.48 ± 0.19 (1.19 to 1.86)
*F* value	0.534	5.279	1.255	3.145
*P* value	0.588	0.007	0.291	0.049

IOL: intraocular lens, log(s): straylight parameter.

## Abbreviations

IOLs: Intraocular lens
PCO: Posterior capsular opacification
ESD: Estimated standard deviation
*Q*: Quality factor.
